# Zonulina e Presepsina Poderiam ser Biomarcadores e Alvos Terapêuticos para Miocardite Aguda?

**DOI:** 10.36660/abc.20230017

**Published:** 2023-07-19

**Authors:** Kenan Toprak, Mehmet Inanır, Tolga Memioğlu, Mustafa Kaplangoray, Ali Palice, Mustafa Begenc Tascanov

**Affiliations:** 1 Harran University Faculty of Medicine Department of Cardiology Sanliurfa Turquia Harran University Faculty of Medicine – Department of Cardiology, Sanliurfa – Turquia; 2 Abant Izzet Baysal University Hospital Bolu Turquia Abant Izzet Baysal University Hospital – Cardiology, Bolu – Turquia; 3 Sanliurfa Mehmet Akif Inan Training and Research Sanliurfa Turquia Sanliurfa Mehmet Akif Inan Training and Research – Cardiology, Sanliurfa – Turquia

**Keywords:** Biomarcadores, Miocardite, Permeabilidade

## Abstract

**Fundamento:**

O diagnóstico de miocardite aguda geralmente é feito diante de parâmetros clínicos e laboratoriais, podendo, por vezes, ser confundido com doenças que compartilham de características clínicas semelhantes, o que dificulta o diagnóstico. Sendo assim, o uso de biomarcadores mais específicos, para além dos clássicos como a troponina, acelerará o diagnóstico. Além disso, esses biomarcadores podem nos ajudar a compreender melhor o mecanismo de desenvolvimento da miocardite e, assim, prever resultados clínicos imprevisíveis.

**Objetivo:**

Este estudo tem como objetivo revelar a possível relação entre permeabilidade intestinal e miocardite aguda.

**Métodos:**

Neste estudo, buscamos avaliar os níveis séricos de zonulina e presepsina em 138 indivíduos consecutivos, incluindo 68 pacientes com miocardite e outros 70 usados como grupo controle, pareados por idade, sexo e fatores de risco cardiovascular. Valores de p < 0,05 foram considerados estatisticamente significativos.

**Resultados:**

Em comparação com o grupo controle, zonulina e presepsina foram significativamente maiores no grupo de pacientes com miocardite (p < 0,001, para todos). Os níveis de zonulina foram positivamente correlacionados com presepsina, pico de CK-MB e níveis máximos de troponina (r = 0,461, p < 0,001; r = 0,744, p < 0,001; r = 0,627, p < 0,001; respectivamente). Na análise de regressão, presepsina e zonulina foram determinadas como preditores independentes para miocardite (OR de 1,002, IC de 95% 1,001-1,003, p = 0,025; OR de 12,331, IC de 95% 4,261-35,689; p < 0,001; respectivamente). O valor preditivo de miocardite aguda de presepsina e zonulina na análise da curva ROC foi estatisticamente significativo (p < 0,001, para ambos).

**Conclusão:**

Este estudo mostrou que a zonulina e a presepsina podem ser biomarcadores para o diagnóstico de miocardite e também podem ser alvos terapêuticos para esclarecer o mecanismo de desenvolvimento da miocardite.

**Figura Central:**
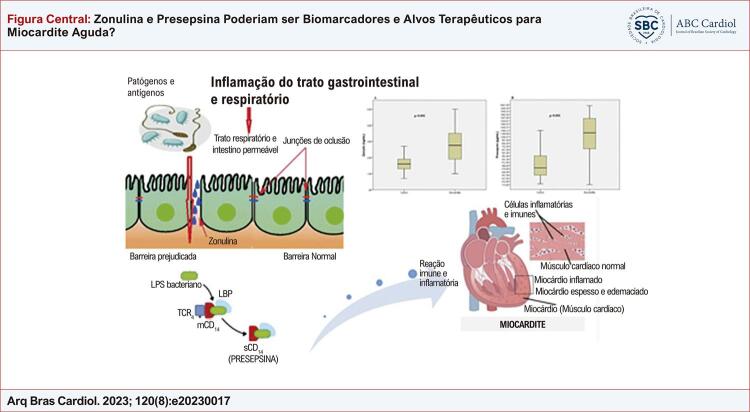
: Zonulina e Presepsina Poderiam ser Biomarcadores e Alvos Terapêuticos para Miocardite Aguda?

## Introdução

A miocardite geralmente se refere às manifestações clínicas e histológicas de uma ampla variedade de processos cardíacos imunopatológicos que podem ocorrer devido à ativação secundária do sistema imunológico após infecções do trato respiratório e gastrointestinais.^1^ Embora tenham sido indicados vários mecanismos no processo de desenvolvimento da doença, sabe-se que, mesmo que o mecanismo exato não seja totalmente compreendido, a doença pode ser causada pelo efeito direto do agente infeccioso, mas é causada principalmente pelo desencadeamento da reação imunológica pós-infecção.^2 - 4^ A miocardite pode ser aguda, subaguda ou crônica e pode envolver áreas focais ou difusas do miocárdio. As manifestações clínicas da miocardite são heterogêneas, variando desde condições assintomáticas até a destruição miocárdica grave por vírus e células imunes, causando choque cardiogênico e arritmias.^5^

A miocardite pode ser causada por uma ampla variedade de agentes microbianos, incluindo vírus, bactérias entéricas e protozoários, bem como por desencadeantes não microbianos, como toxinas e reações de hipersensibilidade.^6^ Entre as causas, infecções virais, especialmente de vírus coxsackie e parvovírus B19 , estão entre as principais causas de miocardite^7^ e, nos últimos anos, o vírus da Covid-19 e a vacina contra a Covid-19 têm sido intimamente associados à miocardite.^8 , 9^ Além disso, bactérias enteropatogênicas como salmonela, shigella e campylobacter são algumas das principais causas de miocardite identificadas em séries de casos.^10^

O diagnóstico da miocardite aguda continua sendo complexo e desafiador na prática de rotina, uma vez que pode ser clinicamente confundido com apresentações clínicas semelhantes, como infarto do miocárdio, vasoespasmo coronariano, amiloidose cardíaca e cardiomiopatia hipertrófica.^1^ O diagnóstico padrão-ouro atual envolve a demonstração histológica de biópsia endomiocárdica, de acordo com os critérios de Dallas para “inflamação miocárdica” sem necrose miocárdica e isquemia.^3^ No entanto, além da impraticabilidade da biópsia endomiocárdica, a definição de miocardite de acordo com esses critérios demonstra baixa sensibilidade e especificidade. Isso se deve ao erro de amostragem frequentemente relacionado à distribuição focal de lesões histológicas específicas do tecido cardíaco e à variabilidade na interpretação patológica. Além disso, a classificação de Dallas não considera a quantificação e a diferenciação local das células inflamatórias, e a infecção viral e a regulação autoimune nos tecidos cardíacos são ignoradas.^11 , 12^ Por esses motivos, a ressonância magnética cardíaca (RMC) com contraste e parâmetros laboratoriais, juntamente com a avaliação clínica, são os métodos mais frequentemente utilizados no diagnóstico.^6^ No entanto, apresentações clínicas semelhantes, como infarto do miocárdio e vasoespasmo coronariano, complicam a abordagem diagnóstica.^1^ Nesse caso, a determinação de biomarcadores auxiliares mais específicos, para além da isoenzima creatina quinase-MB (CK-MB) e da troponina, que são marcadores frequentemente utilizados de destruição cardíaca em pacientes com sinais clínicos ruins, se torna necessária para orientar diagnósticos e modalidades de tratamento.

Acredita-se que a miocardite e suas complicações sejam imunomediadas. A miocardite aguda geralmente se desenvolve após uma infecção recente do trato respiratório ou queixas gastrointestinais, como gastroenterite.^5^ Nas etiologias infecciosas, quando o agente microbiano entra pelo sistema respiratório ou gastroentérico, ele pode se ligar ao seu receptor específico no coração e causar dano celular e lise. Posteriormente, as partículas celulares liberadas da célula miocárdica após a lesão desencadeiam a autoimunidade na qual o mimetismo molecular desempenha um papel importante, ao mesmo tempo em que causa danos cardíacos.^13^

O envolvimento de patógenos gastrointestinais na etiologia da miocardite aguda nos leva a crer que o aumento da permeabilidade intestinal decorrente da inflamação do trato gastrointestinal pode desempenhar um papel na patogênese da doença. As junções de oclusão (JO) das células epiteliais intestinais desempenham um papel fundamental na prevenção da invasão intestinal por patógenos.^14^ A zonulina (precursor da haptoglobina 2) é o único modulador endógeno especificamente identificado para as JOs.^15^ A zonulina modula a integridade da barreira epitelial, desencadeando uma sinalização em cascata que resulta em fosforilação e deslocamento de proteínas da junção de oclusão, e os enteropatógenos aumentam muito a expressão do gene zonulina.^16^ O aumento da permeabilidade gastrointestinal por um mecanismo mediado por zonulina permite a passagem paracelular de desencadeadores antigênicos da luz intestinal para a mucosa e, eventualmente, para a circulação sistêmica. O aumento da permeabilidade intestinal mediado pela zonulina foi indicado como o mecanismo responsável pela fisiopatologia de algumas doenças autoimunes e hiperinflamatórias crônicas.^17^ Isso mostrou que a regulação positiva da zonulina em indivíduos geneticamente suscetíveis pode levar a doenças imunomediadas, e esse mecanismo da via da zonulina pode desempenhar um papel na fisiopatologia da miocardite, que é principalmente uma entidade imunomediada.^4^ Além disso, buscamos entender o papel da endotoxemia de baixo grau, uma espécie de confirmação do aumento da permeabilidade intestinal, na patogênese da miocardite.^18^ Até onde sabemos, não há dados na literatura avaliando o papel da zonulina, um biomarcador de aumento da permeabilidade intestinal, e da presepsina, um biomarcador de endotoxemia de baixo grau, em pacientes com miocardite. Este estudo teve como objetivo descobrir o papel da zonulina e da presepsina em pacientes com miocardite aguda.

## Métodos

### População do estudo

Neste estudo transversal observacional, foram incluídos 68 pacientes, admitidos em nosso centro entre janeiro de 2021 e março de 2022, diagnosticados com miocardite; como grupo controle, 70 pacientes foram incluídos no estudo, sendo pareados em termos de idade, sexo e fatores de risco cardiovascular. O grupo controle foi selecionado por meio do recrutamento consecutivo de voluntários saudáveis, pareados por idade, sexo e doença cardiovascular, que foram admitidos no hospital para exames de rotina. Pacientes com suspeita de infarto do miocárdio, suspeita de angina vasoespástica, doença inflamatória crônica, doença autoimune, infecção ativa, insuficiência renal e hepática avançadas e história de câncer foram excluídos do estudo. A biópsia endomiocárdica não é realizada em nossa clínica para pacientes com miocardite limítrofe caracterizada apenas por infiltrados inflamatórios, sem evidência de necrose de miócitos, e, portanto, nossa população de pacientes consistia apenas de pacientes com necrose de miócitos confirmada por biomarcadores cardíacos.

### Protocolo de estudo e definições

Os pacientes foram divididos em dois grupos: aqueles com miocardite aguda e o grupo controle. Os casos de miocardite com início dos sintomas no mês anterior foram considerados miocardite aguda.^19^ A inflamação cardíaca foi confirmada pela ressonância magnética cardíaca (RMC) com contraste em todos os pacientes. A coronariografia foi realizada para excluir causas isquêmicas nos pacientes que estavam no meio, simulando infarto do miocárdio.

### Análise laboratorial

As análises de sangue foram feitas a partir de amostras de sangue venoso dos pacientes e do grupo controle no momento da admissão no hospital. Amostras de plasma e soro foram obtidas após centrifugação a 2750 × g por 10 minutos. Análises bioquímicas de rotina, hemograma completo e testes de coagulação foram realizados em amostras de sangue. As amostras de soro para análise de presepsina e zonulina foram congeladas e mantidas a -20 °C até o teste. Os níveis de presepsina e zonulina foram medidos por meio de kits comerciais de Ensaio de Imunoabsorção Enzimática (ELISA) (kit ELISA de presepsina humana: Abbexa, Cambridge, Reino Unido; Kit ELISA de zonulina humana: Immundiagnostik AG, Bensheim, Alemanha). Os resultados são apresentados em pg/mL para presepsina e ng/mL para zonulina. Para valores de pico de CK-MB e troponina, amostras de sangue foram coletadas a cada 12 horas durante a internação.

### Análise estatística

O Statistical Program for Social Sciences 20 (IBM SPSS, Chicago, IL, EUA) foi utilizado para todos os cálculos estatísticos. O teste de Kolmogorov-Smirnov foi usado para determinar se os dados correspondiam à distribuição normal. As variáveis contínuas que se ajustaram à distribuição normal foram expressas como média ± desvio padrão (DP) e as que não se ajustaram à distribuição normal foram expressas como mediana com intervalo interquartil (IIQ). As variáveis categóricas são expressas como um número de pessoas por cento (n, %). As comparações entre pacientes com miocardite e grupo controle foram analisadas usando o teste U de Mann-Whitney, teste t independente e análise de variância (ANOVA) de uma via, conforme apropriado. A análise do teste *post hoc* de Bonferroni foi realizada para entender completamente as diferenças de grupo na ANOVA. O teste *post hoc* de Kruskal-Wallis e Brown-Forsythe foi usado para comparações de grupos múltiplos que não se ajustavam à distribuição normal. O teste Qui-quadrado foi aplicado para variáveis categóricas. O coeficiente de correlação de Pearson foi usado para determinar a relação entre o nível de zonulina e os níveis de presepsina, CK-MB e troponina. Análises de regressão logística univariada e multivariada foram realizadas para determinar os preditores independentes de miocardite. As variáveis basais com significância considerável (p < 0,05) pela análise de regressão logística univariada foram incluídas na análise de regressão logística multivariada. A análise da Curva Característica de Operação do Receptor (Curva ROC) foi realizada para identificar preditores de miocardite aguda e comparar os valores preditivos de zonulina e presepsina com outros biomarcadores. O tamanho da amostra necessário para este estudo foi calculado com um pacote R de “poder” usando alfa = 0,05, poder = 0,80 e o tamanho do efeito estimado calculado a partir de relatórios anteriores e o tamanho mínimo da amostra necessário para este estudo foi de 48. Portanto, o número de pacientes em nosso estudo foi suficiente. Também calculamos uma análise de poder *post hoc* para nossa pesquisa, que resultou em 99% do poder. Valores de p bicaudais < 0,05 foram considerados estatisticamente significativos.

## Resultados

**Figura 1:**
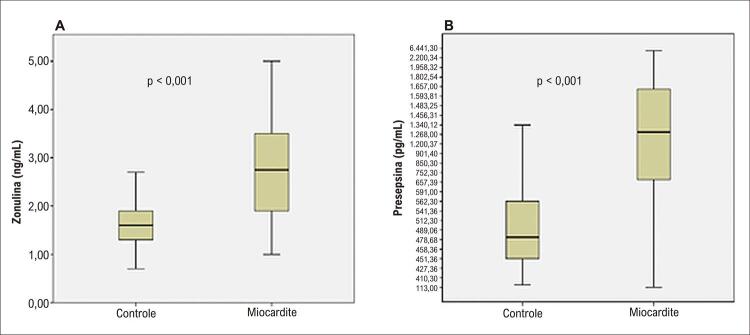
– Níveis de zonulina (A) e presepsina (B) em pacientes com miocardite em comparação com o grupo controle.

**Figura 2:**
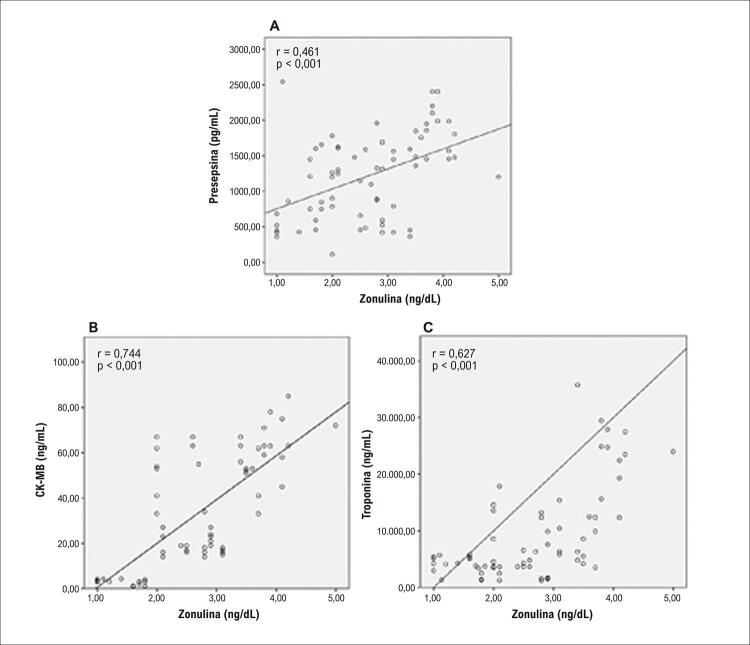
– Correlação positiva entre Zonulina e Presepsina (A), Zonulina e CK-MB (B), Zonulina e Troponina (C) em pacientes com miocardite.

Um total de 68 pacientes diagnosticados com miocardite aguda e 70 voluntários saudáveis pareados por idade, sexo e fatores de risco cardiovascular participaram do estudo. A média de idade dos pacientes foi de 24,75 ± 6,86, sendo 43 (63,2%) do sexo masculino. Não houve diferença entre as características demográficas básicas dos pacientes e do grupo controle ( Tabela 1 ). O histórico de COVID-19 ou vacinação contra COVID-19 nos últimos seis meses foi significativamente maior no grupo de miocardite do que no grupo controle. Os níveis de proteína C-reativa (PCR), fibrinogênio, pico de CK-MB e pico de troponina-I foram significativamente maiores no grupo de miocardite do que no grupo controle. Os níveis de zonulina e presepsina foram estatisticamente maiores no grupo de pacientes em comparação com o grupo controle ( Figura 1 ). Dividimos os pacientes em 3 grupos, de acordo com seus quartis de zonulina (T) (T1 [zonulina ≤ 1,50], T2 [1,50 < zonulina ≤ 2,90], T3 [zonulina > 2,90]) ( Tabela 2 ). Não houve diferença estatisticamente significativa entre os grupos quanto a idade, sexo e tabagismo. O grupo 1 foi tomado como referência na comparação dos grupos. Os pacientes do grupo 3 apresentaram menor fração de ejeção do ventrículo esquerdo (FEVE) do que os grupos 1 e 2, e apresentaram mais distúrbios do ritmo cardíaco. Aqueles que relataram queixas gastrointestinais nas quatro semanas anteriores à internação foram significativamente mais comuns no grupo 3 do que nos grupos 1 e 2. Além disso, os valores de pico de CK-MB, pico de troponina-I e presepsina foram significativamente maiores no grupo 3 em comparação com os outros grupos. No grupo de pacientes, os níveis de zonulina foram positivamente correlacionados com presepsina, pico de CK-MB e pico de troponina ( Figura 2 ). Presepsina e zonulina foram preditores independentes de miocardite aguda na análise de regressão logística binária multivariada ( Tabela 3 ).

**Tabela 1:** – Distribuição das características demográficas, clínicas e laboratoriais básicas de pacientes com miocardite e grupo controle

Variáveis	Pacientes com miocardite (n = 68)	Grupo controle (n = 70)	p

Dados demográficos e história médica			

Idade, anos	24,75 ± 6,86	25,43 ± 5,38	0,495
Gênero, masculino, n (%)	43 (63,2)	44 (62,8)	0,552
IMC, Kg/m^2^	27,3 ± 1,8	27,1 ± 2,1	0,513
Tabagismo, n (%)	14 (20,5)	8 (11,4)	0,142
FEVE, (%)	60,0 (55,0-60,0)	60,0 (55,7-61,0)	0,315
História de COVID-19 ou vacina contra a COVID-19 nos últimos seis meses	26 (38,2)	14 (20,0)	0,015*

**Resultados Laboratoriais**			
Hemoglobina, (mg/dL)	14,72 ± 3,59	14,60 ± 1,74	0,480
Hematócrito, (%)	43,66 ± 6,43	44,84 ± 5,68	0,372
Leucócitos, (x1000/mm^3^)	10,9 (9,1-13,6)	10,9 (9,0-14,9)	0,631
Linfócitos, (x1000/mm^3^)	2,00 (1,50-2,69)	2,10 (1,56-2,72)	0,599
Monócitos, (x1000/mm^3^)	0,68 (0,49-0,89)	0,60 (0,42-0,80)	0,280
Neutrófilos, (x1000/mm^3^)	7,77 (5,22-11,00)	7,70 (5,37-11,0)	0,868
PG, (mg/dL)	103,00 (127,00-71,40)	110,00 (93,75-159,75)	0,100
Creatinina, (mg/dL)	0,84 ± 0,31	0,85 ± 0,21	0,176
Ácido úrico, (mg/dL)	5,05 (5,98-6,47)	5,25 (4,20-6,10)	0,858
Albumina, (mg/dL)	4,35 ± 0,45	4,38 ± 0,34	0,560
LDH, U/L	266 (216-409)	251 (213-356)	0,651
Triglicerídeos, (mg/dL)	149,5 (95,7-213,7)	147,5 (102,2-237,0)	0,519
TC, (mg/dL)	187,0 (147,7-221,5)	181,5 (137,0-221,0)	0,368
HDL-C, (mg/dL)	32,0 (27,3-38,0)	34,0 (28,0-42,0)	0,410
LDL-C, (mg/dL)	119,8 (87,2-146,5)	106,3 (84,7-126,1)	0,174
CRP, (mg/dL)	0,94 (1,86-2,19)	0,40 (0,14-0,80)	0,002**
TSH, uIU/mL	1,23 (0,74-1,88)	1,46 (1,08-2,02)	0,160
FT_3_, ng/mL	3,34 (3,24-3,60)	3,04 (2,68-3,17)	0,068
FT_4,_ ng/mL	1,15 (1,03-1,29)	1,10 (1,01-1,34)	0,937
e-GFR, (ml/min)	105,0 (85,0-117,0)	107,0 (100,2-114,2)	0,641
RDW, fL	12,70 (12,30-13,42)	12,75 (11,45-13,77)	0,642
MPV, fL	8,19 (7,42-9,32)	7,98 (7,20-8,67)	0,141
Bilirrubina total, (mg/dL)	0,60 (0,48-0,80)	0,60 (0,50-0,92)	0,738
Fósforo, (mg/dL)	3,10 (2,50-3,70)	3,10 (2,32-3,70)	0,934
Cálcio, (mg/dL)	9,20 (8,78-9,60)	9,20 (9,00-9,70)	0,809
ALT, (U/L)	28,00 (20,25-41,75)	32,0 (22,25-44,00)	0,496
AST, (U/L)	35,00 (24,00-51,00)	34,00 (25,50-49,00)	0,790
ALP, (U/L)	79,0 (65,0-86,0)	83,0 (73,0-97,0)	0,053
GGT, (U/L)	23,50 (16,00-39,50)	26,00 (18,00-45,00)	0,289
Contagem de plaquetas, (x1000/mm^3^)	283,0 (230,0-333,0)	254,5 (219,7-318,0)	0,642
Fibrinogênio, (mg/dL)	429,2 (475,0-283,0)	283,0 (228,7-440,7)	0,004**
Presepsina, (pg/mL)	1.257,9 (1197,8-602,9)	479,1 (451,3-569,0)	<0,001**
Zonulina, (ng/mL)	2,75 (1,85-3,50)	1,60 (1,30-1,92)	<0,001**
Pico de CK-MB, (ng/mL)	23,50 (6,72-57,50)	3,00 (2,00-6,22)	<0,001**
Pico de Troponina-I, (pg/mL)	4.660,0 (352,5-12458,0)	57,0 (42,25-1130,8)	<0,001**

Salvo indicação em contrário, os valores são média ± DP, n (%) ou mediana (intervalo interquartil). Não houve comparações estatisticamente significativas após análise com o teste t de Student. *p <0,05 vs. controle: Significativo após análise do teste qui-quadrado. **p <0,05 vs. controle: Significativo após análise do teste U de Mann-Whitney. ALP: fosfatase alcalina; ALT: Alanina Aminotransferase; AST: Aspartato aminotransferase; CT: colesterol total; e-GFR: taxa de filtração glomerular estimada; FEVE: fração de ejeção do ventrículo esquerdo; FT3: T3 livre; FT4: T4 livre; GGT: gama glutamil transferase; HDL-C: colesterol de lipoproteína de alta densidade; IMC: índice de massa corporal; LDH: lactato desidrogenase; LDL-C: colesterol de lipoproteína de baixa densidade; PCR: Proteína C-reativa; PG: glicose plasmática; RDW: largura de distribuição das hemácias; TSH: hormônio estimulante da tireoide; VPM: volume plaquetário médio; WBC: glóbulos brancos.

**Tabela 2:** – Distribuição das características demográficas e clínicas de pacientes com miocardite de acordo com os grupos de zonulina

Variáveis	*Grupo 1 n = 22	Grupo 2 n = 23	Grupo 3 n = 23	p

Dados demográficos e história médica				
Idade, anos	22,91 ± 6,43	27,17 ± 6,42	24,09 ± 7,24	0,095
Gênero, masculino, n (%)	15 (68,2)	13 (56,5)	15 (65,2)	0,699
Tabagismo, n (%)	4 (18,2)	5 (21,7)	5 (21,7)	0,944
História de COVID-19 ou vacina contra a COVID-19 nos últimos seis meses	3 (13,6)	7 (30,4)	16 (69,6)	<0,001**

**Ecocardiografia e ECG**				
FEVE, (%)	59,5 ± 2,0	57,9 ± 7,4	49,7 ± 13,7	0,001**
Distúrbios do ritmo cardíaco	1 (4,5)	3 (13,0)	8 (34,7)	<0,001**
**Sintomas nas últimas quatro semanas, n (%)**				
Queixas respiratórias	7 (31,8)	9 (39,1)	9 (39,1)	0,251
Queixas gastrointestinais	6 (27,2)	7 (30,4)	12 (52,2)	<0,001**
Queixas respiratórias e gastrointestinais	2 (9,0)	1 (4,3)	2 (8,7)	0,324
Nenhuma	9 (40,9)	6 (26,1)	-	-
**Resultados Laboratoriais**				
CRP, (mg/dL)	1,42 (0,68-2,17)	1,80 (0,85-2,75)	2,35 (1,22-3,48)	0,369
Fibrinogênio, (mg/dL)	357,2 (295,9-418,5)	407,8 (352,5-463,0)	399,4 (369,3-429,5)	0,124
Pico de CK-MB, (ng/mL)	13,86 (4,21-23,51)	29,86 (19,86-33,87)	56,34 (48,86-63,83)	<0,001***
Pico de Troponina-I, (pg/mL)	2.573,9 (3.104,2-4.362,2)	6.495,0 (4.256,6-8.733,3)	26.737,8 (2.973,8-50.501,8)	<0,001***
Presepsina, (pg/mL)	942,8 (682,5-1.203,1)	1.139,8 (941,4-1.338,2)	1.561,5 (1.306,5-1.816,6)	0,001***

Salvo indicação em contrário, os valores são média ± DP, n (%) ou mediana (intervalo interquartil). * Grupo 1 usado como referência. **p<0,05 vs. controle: Significativo após análise do teste One-Way ANOVA. ***p <0,05 vs. controle: Significativo após análise do teste de Kruskal-Wallis. CK-MB: creatina quinase-MB; ECG: eletrocardiografia.

**Tabela 3:** – Preditores independentes de miocardite aguda

	Análises multivariadas de regressão logística		

	Nagelkerke R^**2**^ na etapa final = 0,643		

	Odds ratio	CI de 95%	Valor de p
Zonulina	12,331	4,261-35,689	<0,001*
Presepsina	1,001	1,000-1,002	0,025*

* Valor-p significativo. Variáveis inseridas: Proteína C-reativa, História de COVID-19 ou vacinação contra COVID-19 nos últimos seis meses, Fibrinogênio, Queixas gastrointestinais, Distúrbios do ritmo cardíaco, Fração de ejeção do ventrículo esquerdo, Presepsina, Zonulina.

**Figura 3:**
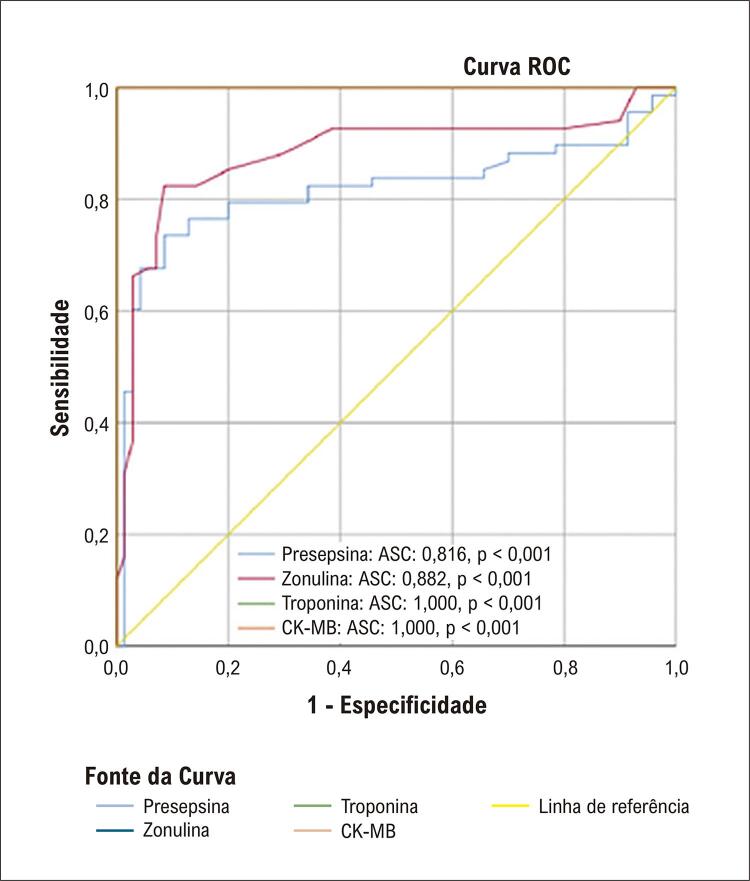
– Curva Característica de Operação do Receptor (ROC) de presepsina, zonulina, troponina e CK-MB como preditores de miocardite aguda. Como a ASC da CK-MB e a ASC da troponina são 1,0, elas se sobrepõem.

Quando a análise da curva Característica de Operação do Receptor (ROC) foi realizada, o valor de corte ideal de presepsina para prever miocardite aguda é ≥ 584,13; esse valor foi preditivo para miocardite aguda com sensibilidade de 79,4% e especificidade de 80% e zonulina ≥ 1,85; sendo preditivo para miocardite aguda com sensibilidade de 82% e especificidade de 91% ( Figura 3 ). Além disso, as curvas ROC foram comparadas para identificar se houve benefício adicional do uso de presepsina e zonulina sobre CK-MB e Troponina-I ( Figura 3 ). Quando a presepsina e a zonulina foram comparadas, elas não se mostraram superiores entre si como preditores de miocardite aguda (p = 0,105).

## Discussão

Atualmente, o diagnóstico e o tratamento da miocardite permanecem desafiadores entre os médicos devido à incapacidade de prever complicações de longo prazo, como a cardiomiopatia dilatada. No entanto, os mecanismos que desencadeiam a miocardite ainda não são totalmente compreendidos. Neste estudo, buscamos compreender o papel da zonulina, que mostra permeabilidade intestinal aumentada, e da presepsina, um biomarcador de endotoxemia de baixo grau, na fisiopatologia da miocardite e se eles podem ser biomarcadores de miocardite aguda. Em nosso estudo, quantidades aumentadas de zonulina e presepsina, em comparação com o grupo controle, mostraram que o aumento da permeabilidade intestinal pode exercer um papel desencadeador importante na patogênese da miocardite. Sendo assim, zonulina e presepsina podem ser biomarcadores úteis para o diagnóstico de miocardite aguda.

O diagnóstico de miocardite aguda continua sendo um desafio para os médicos devido a apresentações clínicas semelhantes em vários distúrbios cardiovasculares, como infarto do miocárdio, vasoespasmo coronariano, amiloidose cardíaca e cardiomiopatia hipertrófica.^5^ Se o agente infeccioso for rapidamente eliminado e o processo inflamatório for encerrado, a doença é curada com apenas pequenas alterações no miocárdio. Esses pacientes geralmente se recuperam completamente em semanas ou meses. Por outro lado, quando o diagnóstico e o tratamento são iniciados tardiamente, a infecção viral não é eliminada e a resposta imune antiviral é diminuída, podendo ocorrer consequências clínicas irreversíveis que podem resultar em cardiomiopatia dilatada. Portanto, os dados disponíveis contestam a necessidade de identificar pacientes em um estágio inicial e ainda reversível de doença cardíaca associada a vírus.^20^ Embora a biópsia endomiocárdica (BEM) seja o diagnóstico padrão-ouro, o uso da BEM varia muito, dependendo do médico e do quadro clínico dos pacientes. Embora a BEM não possa ser aplicada em todos os centros para diagnosticar miocardite, a sensibilidade da BEM para detectar miocardite é bastante baixa.^11 , 12^ Embora o uso de RMC para pacientes com miocardite tenha aumentado recentemente, a RMC não pode ser realizada em todos os centros, nem pode determinar a extensão da inflamação miocárdica, e a avaliação depende, em grande parte, da experiência clínica. Embora a RMC tenha se tornado útil na obtenção não invasiva de informações preliminares precisas e confiáveis, os dados disponíveis sobre a precisão diagnóstica da RMC são atualmente limitados.^13 , 21^

Muitos casos de miocardite passam despercebidos devido a apresentações assintomáticas ou sintomas inespecíficos. Sendo assim, os pacientes incluídos em nosso estudo, em sua maioria, chegaram ao pronto-socorro com sintomas inespecíficos e marcadores cardíacos elevados, como creatina quinase (CK-MB) e troponina. Embora a CK-MB seja um biomarcador preferencial para miocardite, a sensibilidade da creatina quinase no diagnóstico tardio é baixa, uma vez que os estudos mostram que ela cai consistentemente para níveis basais nos primeiros 3-5 dias.^22^ A confiabilidade da medição dos níveis de biomarcadores cardíacos em pacientes com a suspeita de miocardite é baixa, mas ainda ajudam a confirmar o diagnóstico de miocardite.^23^ Marcadores sorológicos inespecíficos de inflamação, como fibrinogênio e proteína C-reativa, podem estar elevados em casos de miocardite suspeita. Ainda assim, os níveis normais desses testes falham em excluir uma resposta inflamatória no miocárdio e, assim, testes sorológicos convencionais e biomarcadores cardíacos para miocardite têm baixo valor prognóstico no diagnóstico de miocardite.^24^ Devido a todas essas desvantagens, é importante usar biomarcadores que possam ser prognóstico para miocardite, além de marcadores convencionais no diagnóstico e tratamento precoces. A zonulina e a presepsina podem ser biomarcadores promissores neste campo.

Pacientes com miocardite geralmente relatam problemas respiratórios ou gastrointestinais recentes sem sintomas inespecíficos aparentes.^5^ Ao examinarmos as causas da miocardite, encontramos, principalmente, vírus enteropatogênicos, como coxsackievirus, parvovírus B19, adenovírus ou COVID-19, e as causas bacterianas geralmente ocorrem após gastroenterite de bactérias enteropatogênicas, como salmonela, shigella e campylobacter.^7 - 10^ Essa situação sugere que a permeabilidade intestinal prejudicada por qualquer motivo pode desempenhar um papel na patogênese da miocardite por causar translocação viral.

As junções de oclusão são importantes na prevenção da invasão de patógenos para dentro das células epiteliais intestinais e, consequentemente, da circulação sanguínea e linfática.^14^ A zonulina é o único modulador fisiológico conhecido das junções de oclusão intercelulares, e a perda da função de barreira devido à regulação positiva da zonulina leva a um fluxo descontrolado de antígenos dietéticos e microbianos na circulação sanguínea e linfática através da submucosa, e este mecanismo tem sido implicado na patogênese de diversas doenças inflamatórias crônicas e autoimunes.^25 , 26^ A autoimunidade desempenha um papel importante na fisiopatologia do desenvolvimento da miocardite.^4 , 27^ Além de autoimunidade, a ativação do complemento também desempenha um papel na patogênese da miocardite.^28^ Estudos demonstraram que a zonulina ativa a via do complemento,^29^ o que sugere que a ativação do complemento mediado pela zonulina pode desempenhar um papel no desenvolvimento da miocardite.

Sugerimos que o aumento da permeabilidade epitelial do trato respiratório^29^ e gastrointestinal^15 - 17^ mediado por zonulina, por várias razões, pode ser a patogênese desencadeadora do desenvolvimento de miocardite, assim como em outras doenças inflamatórias e autoimunes.^4 , 25 , 26^

O Coxsackie B3 e o parvovírus B19 são as causas mais comuns de miocardite em todo o mundo.^3^ Os Coxsackievirus invadem a partir dos enterócitos ligando-se ao receptor de adenovírus e coxsackievirus (CAR, sigla em inglês para *coxsackievirus and adenovirus receptor* ) localizado em complexos de junções de oclusão do intestino.^30 , 31^ Isso pode sugerir que patógenos como coxsackievirus, que muitas vezes desempenham um papel na patogênese da miocardite, desencadeiam o desenvolvimento da miocardite interrompendo a função das junções de oclusão via receptores CAR localizados em complexos de junções de oclusão, regulados via zonulina no epitélio intestinal, e este mecanismo pode explicar o possível aumento da permeabilidade intestinal após infecções por coxsackievirus. Estudos demonstraram que o parvovírus B19, uma das causas mais comuns de miocardite, coloniza células epiteliais intestinais e as usa como reservatório.^32^ O parvovírus B19 foi considerado responsável pela síndrome da fadiga crônica por permanecer em um reservatório no intestino^32^ e o aumento da permeabilidade intestinal em pacientes com síndrome da fadiga crônica tem sido enfatizado por seu papel importante na patogênese da doença.^33^ Isso pode esclarecer a associação do aumento da permeabilidade intestinal com o parvovírus B19 em pacientes com miocardite. Além de patógenos clássicos, como parvovírus B19 e coxsackievírus, casos de miocardite relacionados a COVID-19 começaram a ser observados recentemente diante da epidemia de COVID-19.^8 , 9^ Embora muitos mecanismos fisiopatológicos tenham sido sugeridos no desenvolvimento de miocardite devido à COVID-19, o principal fator desencadeante da doença, ainda não é conhecido.^8^ Além de afetar diversos sistemas, a COVID-19 afeta o sistema gastrointestinal com frequência.^34^ Ao mesmo tempo, estudos descobriram que a permeabilidade intestinal é aumentada em pessoas com COVID -19, e altos níveis de zonulina, um biomarcador de permeabilidade intestinal, foram encontrados nesses pacientes.^35^ Embora o efeito da permeabilidade intestinal não tenha sido demonstrado em pacientes com miocardite por COVID-19, os níveis mais altos de zonulina observados em pacientes com miocardite com história recente de COVID-19, como ocorre em nosso estudo, pode corroborar nossa ideia de que a COVID-19 desencadeia o desenvolvimento de miocardite por aumentar a permeabilidade intestinal. Além disso, embora observado com menor frequência do que os agentes virais, o desenvolvimento de miocardite após gastroenterite de bactérias enteropatogênicas indica que a permeabilidade intestinal prejudicada pode estar associada ao desenvolvimento de miocardite.^36^

Estudos demonstraram que os lipopolissacarídeos (endotoxinas) aumentam a permeabilidade intestinal.^18^ A presepsina (um grupo solúvel de diferenciação subtipo 14 [CD14]) é um marcador confiável e indireto de endotoxemia.^37^ Ela aumenta na sepse bacteriana e também em infecções virais como a COVID-19.^38^ Tudo isso mostra que a presepsina pode ser um biomarcador indireto que pode mostrar um aumento da permeabilidade intestinal.

Em nosso estudo, a correlação de altos níveis de zonulina com picos de CK-MB e picos de troponina-I indica que uma maior carga de antígeno desencadeia maior destruição cardíaca à medida que a permeabilidade intestinal aumenta. O fato de os níveis de zonulina e presepsina estarem elevados em pacientes com miocardite pode ser epifenomenal ou indicar uma interação complexa, com um papel no aumento da permeabilidade intestinal. O aumento da permeabilidade intestinal, confirmado pelos níveis de zonulina, pode levar a mais bacteremia e viremia, o que pode ser explicado pelo aumento da presepsina em correlação com os níveis de zonulina. Além disso, ao considerarmos o papel da zonulina na patogênese da miocardite, ela pode ser um potencial biomarcador, bem como um alvo terapêutico em casos graves de miocardite, como em outras doenças inflamatórias.^39^ Os vírus entéricos utilizam micróbios intestinais para replicação e transmissão,^40^ o que pode explicar por que o aumento da permeabilidade intestinal na enterite gastrointestinal bacteriana causa transmissão de vírus entéricos e predispõe ao desenvolvimento de miocardite. O fato de a presepsina e a zonulina estarem elevadas simultaneamente em casos de miocardite corrobora essa visão.

Como resultado, levantamos a hipótese de que o aumento da permeabilidade do sistema gastrointestinal pode ser um dos principais mecanismos desencadeantes no desenvolvimento da miocardite. A zonulina e a presepsina podem ser usadas como biomarcadores, além dos biomarcadores convencionais, quando avaliados pela clínica. Isso visa o manejo precoce de modalidades de diagnóstico e tratamento em miocardite, em que ferramentas de diagnóstico padrão-ouro, como BEM e RMC, não estão disponíveis.

Se essa relação for claramente demonstrada, o diagnóstico e o tratamento precoces podem ajudar a prevenir complicações crônicas da miocardite, como a cardiomiopatia dilatada. Além disso, em casos com apresentações clínicas semelhantes, o uso desses dois biomarcadores em adição aos biomarcadores tradicionais é uma opção mais econômica na prática clínica, uma vez que reduz a aplicação de métodos diagnósticos caros, como BEM, RMC e coronariografia, que são usados para o diagnóstico e exclusão de miocardite aguda e requerem um conhecimento especializado para a sua aplicação e interpretação de resultados.

Com base nesses achados, estudos mais extensos nessa área podem esclarecer a patogênese desencadeante da miocardite e auxiliar no desenvolvimento de novos modelos farmacológicos para reduzir a probabilidade de desfechos clínicos ruins, como a cardiomiopatia dilatada.

### Limitações do estudo

Nosso estudo apresentou algumas limitações. Primeiramente, nosso tamanho de amostra foi relativamente pequeno e tratou-se de um estudo transversal. Em segundo lugar, apenas pacientes com miocardite aguda, com biomarcadores cardíacos elevados, foram incluídos em nosso estudo. A exclusão de pacientes com miocardite limítrofe e subclínica e pacientes com miocardite crônica pode afetar os resultados. Os resultados de PCR ou isolamento dos vírus causadores não puderam ser incluídos no estudo devido à inadequação técnica. Ainda assim, a avaliação combinada da história dos pacientes, resultados de RMC, marcadores biológicos como troponina e CK-MB e métodos invasivos e não invasivos de angiografia coronária foram suficientes para excluir outras causas. Por fim, a biópsia endomiocárdica, ferramenta diagnóstica padrão-ouro, não foi utilizada no diagnóstico, o que pode ter afetado nossos resultados.

## Conclusão

A miocardite pode se apresentar, de forma semelhante à cardiopatia isquêmica, com dor torácica, anormalidades nos eletrocardiogramas e biomarcadores cardíacos elevados, podendo ser confundida com patologias cardíacas que são semelhantes em sintomas e achados laboratoriais, incluindo amiloidose cardíaca e cardiomiopatia hipertrófica. Portanto, casos suspeitos de miocardite permanecem um diagnóstico desafiador para os médicos em termos de apresentação, características e curso. A esse respeito, quando ferramentas diagnósticas padrão-ouro, como BEM e RMC, não estão disponíveis, biomarcadores auxiliares, além de CK-MB e troponina cardíaca, podem ser usados para diagnóstico rápido de casos de miocardite e exclusão de outras causas cardíacas. Além disso, o aumento do trato respiratório e da permeabilidade intestinal pode ser um dos principais mecanismos desencadeantes do desenvolvimento da miocardite. Sendo assim, a zonulina e a presepsina podem ser biomarcadores promissores tanto para o diagnóstico quanto para o tratamento de acompanhamento em pacientes com miocardite.
