# Supplementary material for: Zonulina e Presepsina Poderiam ser Biomarcadores e Alvos Terapêuticos para Miocardite Aguda?
Source: Arq Bras Cardiol. 2023 Jul 19;120(8):e20230017. [Article in Portuguese] doi: 10.36660/abc.20230017 (PMC10464857; doi:10.36660/abc.20230017)
Supplement: Translation

# Translation: Could Zonulin and Presepsin Be Biomarkers and Therapeutic Targets for Acute Myocarditis?

**Authors:** Kenan Toprak, Mehmet Inanır, Tolga Memioğlu, Mustafa Kaplangoray, Ali Palice, Mustafa Begenc Tascanov

**Affiliations:** 1 Turkey Harran University Faculty of Medicine – Department of Cardiology, Sanliurfa – Turkey; 2 Turkey Abant Izzet Baysal University Hospital – Cardiology, Bolu – Turkey; 3 Turkey Sanliurfa Mehmet Akif Inan Training and Research – Cardiology, Sanliurfa – Turkey

**Keywords:** Biomarkers, Myocarditis, Permeability

## Abstract

**Background:**

The diagnosis of acute myocarditis is usually made with clinical and laboratory parameters. This can sometimes be mixed up with diseases that have similar clinical features, making the diagnosis difficult. Therefore, the use of more specific biomarkers, in addition to the classically used biomarkers such as troponin, will accelerate the diagnosis. In addition, these biomarkers may help us to understand the mechanism of myocarditis development and thus predict unpredictable clinical outcomes.

**Objective:**

This study aims to reveal the possible relationship between intestinal permeability and acute myocarditis.

**Methods:**

In this study, we wanted to evaluate serum levels of zonulin and presepsin in 138 consecutive subjects, including 68 patients with myocarditis and another 70 as the control group, matched for age, gender, and cardiovascular risk factors. P-values <0.05 were considered to be statistically significant.

**Results:**

Compared to the control group, zonulin and presepsin were significantly higher in the patient group with myocarditis (p < 0.001, for all). Zonulin levels were positively correlated with presepsin, peak CK-MB, and peak troponin levels (r = 0.461, p < 0.001; r = 0.744, p < 0.001; r = 0.627, p < 0.001; respectively). In regression analysis, presepsin and zonulin were determined as independent predictors for myocarditis (OR 1.002, 95% CI 1.001-1.003, p = 0.025; OR 12.331, 95% CI 4.261-35.689; p < 0.001; respectively). The predictive value of acute myocarditis of presepsin and zonulin in ROC curve analysis was statistically significant (p < 0.001, for both).

**Conclusion:**

This study showed that zonulin and presepsin could be biomarkers that can be used in the diagnosis of myocarditis, and they can also be therapeutic targets by shedding light on the developmental mechanism of myocarditis.

**Central Illustration:** : Could Zonulin and Presepsin Be Biomarkers and Therapeutic Targets for Acute Myocarditis?

## Introduction

Myocarditis generally refers to the clinical and histological manifestations of a wide variety of pathological immune processes in the heart that may occur due to secondary immune system activation after respiratory tract and gastrointestinal infections.^1^ Although various mechanisms have been suggested in the development process of the disease, it is known that although the exact mechanism is not fully understood, the disease may be caused by the direct effect of the infective agent, but it is mostly caused by the triggering of the immune reaction after the infection.^2 - 4^ Myocarditis can be acute, subacute, or chronic and may either involve focal or diffuse areas of the myocardium. The clinical manifestations of myocarditis are heterogeneous, ranging from asymptomatic conditions to severe myocardial destruction by viruses and immune cells causing cardiogenic shock and arrhythmias.^5^

Myocarditis can be caused by a wide variety of microbial agents, including viruses, enteric bacteria, and protozoa, as well as by non-microbial triggers such as toxins and hypersensitive reactions.^6^ Among these causes, viral infections, especially coxsackie virus and parvovirus B19, are among the leading causes of myocarditis,^7^ and in recent years, the Covid-19 virus and the Covid-19 vaccine have been closely associated with myocarditis.^8 , 9^ In addition, enteropathogenic bacteria such as salmonella, shigella and campylobacter are some of the major causes of myocarditis in case series.^10^

Acute myocarditis remains a complex and challenging diagnosis in routine practice, as it can be clinically mixed up with similar clinical presentations such as myocardial infarction, coronary vasospasm, cardiac amyloidosis and hypertrophic cardiomyopathy.^1^ The current gold standard diagnosis involves histological demonstration of endomyocardial biopsy according to the Dallas criteria for “myocardial inflammation” without myocardial necrosis and ischemia.^3^ However, both endomyocardial biopsy is impractical and the definition of myocarditis according to these criteria shows poor sensitivity and specificity. This is due to sampling error often related to the focal distribution of specific histological lesions from heart tissue, and variability in pathological interpretation. Also, the Dallas classification does not consider the local quantification and differentiation of inflammatory cells, and viral infection and autoimmune regulation in heart tissues are ignored in this classification.^11 , 12^ For these reasons, contrast-enhanced cardiac magnetic resonance imaging (cMRI) and laboratory parameters are the most frequently used methods in the diagnosis, together with the clinical diagnosis.^6^ However, similar clinical presentations, such as myocardial infarction and coronary vasospasm, complicate the diagnostic approach.^1^ In this case, the determination of more specific auxiliary biomarkers in addition to creatine kinase-MB isoenzyme (CK-MB) and troponin, which are frequently used markers of cardiac destruction in patients with faint clinical signs, increases the necessity for guiding diagnoses and treatment modalities.

Myocarditis and its complications are believed to be immune-mediated. Acute myocarditis usually develops after a recent respiratory tract infection or gastrointestinal complaints such as gastroenteritis.^5^ In infectious etiologies, when the microbial agent enters through the respiratory or gastroenteric system, it may bind to its specific receptor in the heart and cause cell damage and lysis. Subsequently, cellular particles released from the myocardial cell after myocardial injury trigger autoimmunity in which molecular mimicry plays an important role while also causing cardiac damage.^13^

The involvement of gastrointestinal pathogens in the etiology of acute myocarditis led us to believe that increased intestinal permeability as a result of inflammation of the gastrointestinal tract may play a role in the pathogenesis of the disease. Tight junctions (TJ) in intestinal epithelial cells play a key role in preventing intestinal invasion of pathogens in the intestine.^14^ Zonulin (haptoglobin 2 precursor) is the only endogenous modulator specifically identified for TJs.^15^ Zonulin modulates epithelial barrier integrity by triggering a signaling cascade that results in phosphorylation and displacement of tight junction proteins, and enteropathogens greatly increase zonulin gene expression.^16^ Increased gastrointestinal permeability by a zonulin-mediated mechanism allows paracellular passage of antigenic triggers from the intestinal lumen to the mucosa and eventually to the systemic circulation. The zonulin-mediated increase in intestinal permeability in the gastrointestinal tract has been suggested as the mechanism responsible for the pathophysiology of some chronic autoimmune and hyperinflammatory diseases.^17^ This showed that up-regulation of zonulin in genetically susceptible individuals can lead to immune-mediated diseases, and this zonulin pathway mechanism may play a role in the pathophysiology of myocarditis, which is mainly an immune-mediated entity.^4^ In addition, we aimed to uncover the role of low-grade endotoxemia, a kind of confirmation of increased intestinal permeability, in the pathogenesis of myocarditis.^18^ To the best of our knowledge, there is no literature evaluating the role of zonulin, a biomarker of increased intestinal permeability, and presepsin, a low-grade endotoxemia biomarker, in patients with myocarditis. This study aimed to uncover the role of zonulin and presepsin in patients with acute myocarditis.

## Methods

### Study population

In this observational cross-sectional study, 68 patients admitted to our center between January 2021 and March 2022 and diagnosed with myocarditis were included; as a control group, 70 patients were included in the study by matching them in terms of age, gender, and cardiovascular risk factors. The control group was selected by consecutively recruiting healthy volunteers, matched for age, sex, and cardiovascular disease, who were admitted to the hospital for routine examinations. Those with suspected myocardial infarction, suspected vasospastic angina, chronic inflammatory disease, autoimmune disease, active infection, advanced kidney and liver failure, and a history of cancer were excluded from the study. Since we did not perform endomyocardial biopsy in our clinic for borderline myocarditis patients characterized only by inflammatory infiltrates without evidence of myocyte necrosis, our patient population consisted only of patients with myocyte necrosis confirmed by cardiac biomarkers.

### Study protocol and definitions

The patients were divided into two groups: those with acute myocarditis and the control group. Myocarditis cases with symptoms that started within the last month were considered acute myocarditis.^19^ Cardiac inflammation was confirmed by contrast-enhanced cardiac magnetic resonance imaging (cMRI) in all patients. Coronary angiography was performed to exclude ischemic causes in the patients who were in the middle, mimicking myocardial infarction.

### Laboratory analysis

Blood measurements were taken from venous blood samples from the patients and control groups at the time of admission to the hospital. Plasma and serum specimens were obtained after centrifugation at 2750 × g for 10 minutes. Routine biochemistry analyses, complete blood counts and coagulation tests were conducted on blood samples. Serum samples for presepsin and zonulin analysis were frozen and kept at -20 °C until testing. Presepsin and zonulin levels were measured by using commercial Enzyme-Linked Immunosorbent Assay (ELISA) kits (Human presepsin ELISA kit: Abbexa, Cambridge, UK; Human zonulin ELISA kit: Immundiagnostik AG, Bensheim, Germany). Results are presented in pg/mL for presepsin and ng/mL for zonulin. For peak CK-MB and troponin values, blood samples were taken every 12 hours during hospitalization.

### Statistical analysis

The Statistical Program for Social Sciences 20 (IBM SPSS, Chicago, IL, USA) was used for all statistical calculations. The Kolmogorov-Smirnov test was used to determine whether the data matched the normal distribution. Continuous variables that fit the normal distribution were expressed as means ± standard deviation (SD), and those that did not fit the normal distribution were expressed as median with interquartile range (IQR). Categorical variables are expressed as a number of people-percent (n, %). Comparisons between patients with myocarditis and control group were analyzed using the Mann-Whitney U test, independent t-test, and one-way analysis of variance (ANOVA), where appropriate. Bonferroni post hoc test analysis was performed to fully understand group differences in ANOVA. Kruskal-Wallis and then Brown-Forsythe post hoc test were used for multiple group comparisons that did not fit the normal distribution. The Chi-square test was applied to categorical variables. Pearson correlation coefficient was used to determine the relationship between zonulin level and presepsin, CK-MB and troponin levels. Univariate and multivariate logistic regression analyses were performed to determine the independent predictors of myocarditis. Baseline variables with considerable significance (p < 0.05) by univariate logistic regression analyses were included in the multivariate logistic regression analysis. Receiver Operating Characteristic (ROC) curve analysis was performed to identify predictors of acute myocarditis and to compare the predictive values of zonulin and presepsin with other biomarkers. We calculated the required sample size for this study with a “power” R package using alpha=0.05, power=0.80, and the estimated effect size calculated from previous reports and the minimum sample size required for this study was 48. Therefore, the number of patients in our study was sufficient. We also calculated a post hoc power analysis for our research, which resulted in 99% of the power. Two-tailed p-values of < 0.05 were considered to be statistically significant.

## Results

A total of 68 patients diagnosed with acute myocarditis and 70 healthy volunteers matched for age, sex, and cardiovascular risk factors participated in the study. The mean age of the patients was 24.75 ± 6.86, and 43 (63.2%) were male. There was no difference between the basic demographic characteristics of the patients and the control group ( Table 1 ). The history of having COVID-19 disease or being vaccinated against COVID-19 in the last six months was significantly higher in the myocarditis group than in the control group. C-reactive protein (CRP), fibrinogen, peak CK-MB, and peak troponin-I levels were significantly higher in the myocarditis group than in the control group. Zonulin and presepsin levels were statistically higher in the patient group than in the control group ( Figure 1 ). We divided the patients into 3 groups according to their zonulin quartiles (T) (T1 [zonulin ≤ 1.50], T2 [1.50 < zonulin ≤ 2.90], T3 [zonulin > 2.90]) ( Table 2 ). There was no statistically significant difference between groups in terms of age, gender, and smoking. Group 1 was taken as a reference in the comparison of groups. Patients in group 3 had lower left ventricular ejection fraction (LVEF) than groups 1 and 2, and had more rhythm disturbances. Those who described gastrointestinal complaints within the last four weeks of admission were significantly more common in group 3 than in groups 1 and 2. In addition, Peak CK-MB, peak Troponin-I, and presepsin values were significantly higher in group 3 compared to other groups. In the patient group, zonulin levels were positively correlated with presepsin, peak CK-MB, and peak troponin levels ( Figure 2 ). Presepsin and zonulin were found to be independent predictors of acute myocarditis in multivariate binary logistic regression analysis ( Table 3 ).

**Table 1:** – Distribution of basic demographic, clinical and laboratory characteristics of patients with myocarditis and control group

Variables	Patients with myocarditis (n = 68)	Control group (n = 70)	p

Demographics and medical history			
Age, years	24.75 ± 6.86	25.43 ± 5.38	0.495
Gender, male, n (%)	43 (63.2)	44 (62.8)	0.552
BMI, Kg/m^2^	27.3 ± 1.8	27.1 ± 2.1	0.513
Smoking, n (%)	14 (20.5)	8 (11.4)	0.142
LVEF, (%)	60.0 (55.0-60.0)	60.0 (55.7-61.0)	0.315
History of COVID-19 or COVID-19 vaccination in the last six months	26 (38.2)	14 (20.0)	0.015*

**Laboratory Results**			
Hemoglobin, (mg/dL)	14.72 ± 3.59	14.60 ± 1.74	0.480
Hematocrit, (%)	43.66 ± 6.43	44.84 ± 5.68	0.372
WBC, (x1000/mm^3^)	10.9 (9.1-13.6)	10.9 (9.0-14.9)	0.631
Lymphocyte, (x1000/mm^3^)	2.00 (1.50-2.69)	2.10 (1.56-2.72)	0.599
Monocytes, (x1000/mm^3^)	0.68 (0.49-0.89)	0.60 (0.42-0.80)	0.280
Neutrophil, (x1000/mm^3^)	7.77 (5.22-11.00)	7.70 (5.37-11.0)	0.868
PG, (mg/dL)	103.00 (127.00-71.40)	110.00 (93.75-159.75)	0.100
Creatinine, (mg/dL)	0.84 ± 0.31	0.85 ± 0.21	0.176
Uric acid, (mg/dL)	5.05 (5.98-6.47)	5.25 (4.20-6.10)	0.858
Albumin, (mg/dL)	4.35 ± 0.45	4.38 ± 0.34	0.560
LDH, U/L	266 (216-409)	251 (213-356)	0.651
Triglycerides, (mg/dL)	149.5 (95.7-213.7)	147.5 (102.2-237.0)	0.519
TC, (mg/dL)	187.0 (147.7-221.5)	181.5 (137.0-221.0)	0.368
HDL-C, (mg/dL)	32.0 (27.3-38.0)	34.0 (28.0-42.0)	0.410
LDL-C, (mg/dL)	119.8 (87.2-146.5)	106.3 (84.7-126.1)	0.174
CRP, (mg/dL)	0.94 (1.86-2.19)	0.40 (0.14-0.80)	0.002**
TSH, uIU/mL	1.23 (0.74-1.88)	1.46 (1.08-2.02)	0.160
FT_3_, ng/mL	3.34 (3.24-3.60)	3.04 (2.68-3.17)	0.068
FT_4,_ng/mL	1.15 (1.03-1.29)	1.10 (1.01-1.34)	0.937
e-GFR, (ml/min)	105.0 (85.0-117.0)	107.0 (100.2-114.2)	0.641
RDW, fL	12.70 (12.30-13.42)	12.75 (11.45-13.77)	0.642
MPV, fL	8.19 (7.42-9.32)	7.98 (7.20-8.67)	0.141
Total bilirubin, (mg/dL)	0.60 (0.48-0.80)	0.60 (0.50-0.92)	0.738
Phosphorus, (mg/dL)	3.10 (2.50-3.70)	3.10 (2.32-3.70)	0.934
Calcium, (mg/dL)	9.20 (8.78-9.60)	9.20 (9.00-9.70)	0.809
ALT, (U/L)	28.00 (20.25-41.75)	32.0 (22.25-44.00)	0.496
AST, (U/L)	35.00 (24.00-51.00)	34.00 (25.50-49.00)	0.790
ALP, (U/L)	79.0 (65.0-86.0)	83.0 (73.0-97.0)	0.053
GGT, (U/L)	23.50 (16.00-39.50)	26.00 (18.00-45.00)	0.289
Platelet count, (x1000/mm^3^)	283.0 (230.0-333.0)	254.5 (219.7-318.0)	0.642
Fibrinogen, (mg/dL)	429.2 (475.0-283.0)	283.0 (228.7-440.7)	0.004**
Presepsin, (pg/mL)	1.257.9 (1197.8-602.9)	479.1 (451.3-569.0)	<0.001**
Zonulin, (ng/mL)	2.75 (1.85-3.50)	1.60 (1.30-1.92)	<0.001**
Peak CK-MB, (ng/mL)	23.50 (6.72-57.50)	3.00 (2.00-6.22)	<0.001**
Peak Troponin-I, (pg/mL)	4.660.0 (352.5-12458.0)	57.0 (42.25-1130.8)	<0.001**

Unless otherwise stated, values are mean ± SD, n (%), or median (interquartile range). There were no statistically significant comparisons after analysis with Student’s t-test. *p <0.05 vs. control: Significant after analysis Chi-square test. **p <0.05 vs. control: Significant after analysis of Mann-Whitney U test. ALT: Alanine Aminotransferase; AST: Aspartate aminotransferase; ALP: alkaline phosphatase; BMI: body mass index; CRP: C- reactive protein; PG: plasma glucose; GGT: gamma glutamyl transferase; LDH: lactate dehydrogenase; LVEF: left ventricular ejection fraction; e-GFR: estimated glomerular filtration rate; TSH: thyroid stimulating hormone; FT3 : free T3; FT4: free T4; TC: total cholesterol; HDL-C: high-density lipoprotein cholesterol; LDL-C: low-density lipoprotein cholesterol; MPV: mean platelet volume; RDW: red cell distribution width; WBC: white blood cell.

**Figure 1:** – Zonulin (A) and presepsin (B) levels in patients with myocarditis compared to the control group.

**Table 2:** – Distribution of demographic and clinical characteristics of patients with myocarditis according to zonulin groups

Variables	*Group 1 n = 22	Group 2 n = 23	Group 3 n = 23	p

Demographics and medical history				

Age, years	22.91 ± 6.43	27.17 ± 6.42	24.09 ± 7.24	0.095
Gender, male, n (%)	15 (68.2)	13 (56.5)	15 (65.2)	0.699
Smoking, n (%)	4 (18.2)	5 (21.7)	5 (21.7)	0.944
History of COVID-19 or COVID-19 vaccination in the last six months	3 (13.6)	7 (30.4)	16 (69.6)	<0.001**

**Echocardiography and EKG**				
LVEF, (%)	59.5 ± 2.0	57.9 ± 7.4	49.7 ± 13.7	0.001**
Rhythm disturbances	1 (4.5)	3 (13.0)	8 (34.7)	<0.001**
**Symptoms in the last four weeks, n (%)**				
Respiratory tract complaints	7 (31.8)	9 (39.1)	9 (39.1)	0.251
GIS complaints	6 (27.2)	7 (30.4)	12 (52.2)	<0.001**
Respiratory tract and GIS complaints	2 (9.0)	1 (4.3)	2 (8.7)	0.324
None	9 (40.9)	6 (26.1)	-	-
**Laboratory Results**				
CRP, (mg/dL)	1.42 (0.68-2.17)	1.80 (0.85-2.75)	2.35 (1.22-3.48)	0.369
Fibrinogen, (mg/dL)	357.2 (295.9-418.5)	407.8 (352.5-463.0)	399.4 (369.3-429.5)	0.124
Peak CK-MB, (ng/mL)	13.86 (4.21-23.51)	29.86 (19.86-33.87)	56.34 (48.86-63.83)	<0.001***
Peak Troponin-I, (pg/mL)	2.573.9 (3.104.2-4.362.2)	6.495.0 (4.256.6-8.733.3)	26.737.8 (2.973.8-50.501.8)	<0.001***
Presepsin, (pg/mL)	942.8 (682.5-1.203.1)	1.139.8 (941.4-1.338.2)	1.561.5 (1.306.5-1.816.6)	0.001***

Unless otherwise stated, values are mean ± SD, n (%), or median (interquartile range). * Group 1 taken as reference. **p<0.05 vs. control: Significant after analysis of One-Way ANOVA test. ***p <0.05 vs. control: Significant after analysis of Kruskal-Wallis test. EKG: electrocardiography; GIS: gastrointestinal system; CK-MB: creatine kinase- MB.

**Figure 2:** – Positive correlation between Zonulin and Presepsin (A), Zonulin and CK-MB (B), Zonulin and Troponin (C) in patients with myocarditis.

**Table 3:** – Independent predictors of acute myocarditis

	Multivariate logistic regression analyses		

	Nagelkerke R^**2**^ in final step = 0.643		

	Odds ratio	95% CI	p-value
Zonulin	12.331	4.261-35.689	<0.001*
Presepsin	1.001	1.000-1.002	0.025*

* Significant p-value. Entered variables: C- reactive protein, History of COVID-19 or COVID-19 vaccination in the last six months, Fibrinogen, GIS complaints, Rhythm disturbances, Left ventricular ejection fraction, Presepsin, Zonulin.

When Receiver Operating Characteristic (ROC) curve analysis was performed, the optimal cut-off value of presepsin to predict acute myocarditis is presepsin ≥ 584.13; it was predictive for acute myocarditis with 79.4% sensitivity and 80% specificity, and zonulin ≥ 1.85; it was predictive for acute myocarditis with 82% sensitivity and 91% specificity ( Figure 3 ). In addition, the ROC curves were compared to identify if there was an additional benefit of using presepsin and zonulin on CK-MB and Troponin-I ( Figure 3 ). When presepsin and zonulin were compared, they were not found to be superior to each other in terms of predicting acute myocarditis (p = 0.105).

**Figure 3:** – Receiver Operating Characteristics (ROC) curve of presepsin, zonulin, troponin and CK-MB for predicting acute myocarditis. Since the AUC of CK-MB and the AUC of Troponin are 1.0, they overlayed each other.

## Discussion

Today, the diagnosis and treatment of myocarditis remain challenging among clinicians due to the inability to predict long-term complications such as dilated cardiomyopathy. However, the mechanisms that trigger myocarditis are still not fully understood. In this study, we wanted to uncover the role of zonulin, which shows increased intestinal permeability, and presepsin, a low-grade endotoxemia biomarker, in the pathophysiology of myocarditis and whether they can be biomarkers for acute myocarditis. In our study, increased zonulin and presepsin amounts compared to the control group showed that increased intestinal permeability might have a triggering role in the pathogenesis of myocarditis. Therefore, zonulin and presepsin may be useful biomarkers that can be used in the diagnosis of acute myocarditis.

Acute myocarditis remains a diagnostic challenge for clinicians due to similar clinical presentations in various cardiovascular disorders such as myocardial infarction, coronary vasospasm, cardiac amyloidosis, and hypertrophic cardiomyopathy.^5^ If the infectious agent is quickly eliminated and the inflammatory process is terminated, the disease will heal with only minor changes in the myocardium. These patients usually recover completely within weeks or months. Conversely, when diagnosis and treatment are started too late, the viral infection is not eliminated, and the antiviral immune response is diminished, irreversible clinical consequences may be encountered, which may result in dilated cardiomyopathy. Therefore, available data dispute the need to identify patients at an early and still reversible stage of virus-associated heart disease.^20^ Although endomyocardial biopsy (EMB) is the diagnostic gold standard, the use of EMB varies widely depending on the physician and the clinical presentation of the patients. Although EMB cannot be applied in every center for diagnosing myocarditis, the sensitivity of EMB for detecting myocarditis is quite low.^11 , 12^ Although the use of cardiac MRI for myocarditis patients has recently increased, cardiac MRI cannot be performed in every center nor can it determine the extent of myocardial inflammation, and assessment is largely dependent on clinical experience. Although cMRI has become useful in noninvasively obtaining accurate and reliable preliminary information, available data on the diagnostic accuracy of cMRI is currently limited.^13 , 21^

Many cases of myocarditis go undetected because of asymptomatic presentations or nonspecific symptoms. Therefore, the patients included in our study mostly consisted of patients who arrived at the emergency department with nonspecific symptoms and had high cardiac markers such as creatine kinase (CK-MB) and troponin. Although CK-MB is a preferred biomarker for myocarditis, the sensitivity of creatine kinase in late diagnosis is poor because studies show that it consistently drops to basal levels in the early 3-5 days.^22^ The reliability of measuring cardiac biomarker levels in patients with suspected myocarditis is low, but they still help confirm the diagnosis of myocarditis.^23^ Nonspecific serological markers of inflammation, such as fibrinogen and C-reactive protein, may be elevated in the case of suspected myocarditis. Still, normal levels of these tests fail to exclude an inflammatory response in the myocardium, so conventional serological tests and cardiac biomarkers for myocarditis are of poor prognostic value in diagnosing myocarditis.^24^ Because of all these handicaps, it is important to use biomarkers that can be prognostic for myocarditis in addition to conventional markers in early diagnosis and treatment. Zonulin and presepsin may be promising biomarkers in this field.

Patients with myocarditis often report recent respiratory or gastrointestinal issues without apparent nonspecific symptoms.^5^ When we look at the causes of myocarditis, it is mostly caused by enteropathogenic viruses such as coxsackievirus, parvovirus B19, adenovirus, or COVID-19, and it is seen that bacterial causes often occur after gastroenteritis of enteropathogenic bacteria such as salmonella, shigella and campylobacter.^7 - 10^ This situation suggested that impaired intestinal permeability in the gastrointestinal tract for any reason may play a role in the pathogenesis of myocarditis by causing viral translocation.

Tight junctions are important in preventing pathogen invasion in intestinal epithelial cells and thus into blood and lymph circulation.^14^ Zonulin is the only known physiological modulator of intercellular tight junctions, and loss of barrier function due to upregulation of zonulin leads to an uncontrolled flow of dietary and microbial antigens into blood and lymph circulation through the submucosa, and this mechanism has been implicated in the pathogenesis of many chronic inflammatory and autoimmune diseases.^25 , 26^ Autoimmunity plays an important role in the pathophysiology of myocarditis development.^4 , 27^ In addition to autoimmunity, complement activation also plays a role in the pathogenesis of myocarditis.^28^ Studies have shown that zonulin activates the complement pathway.^29^ This suggests that zonulin-mediated complement activation may play a role in the development of myocarditis.

We suggest that the zonulin-mediated increase in epithelial permeability in respiratory^29^ and gastrointestinal tract^15 - 17^ for various reasons may be the pathogenesis that triggers the development of myocarditis, as in other inflammatory and autoimmune diseases.^4 , 25 , 26^

Coxsackie B3 and parvovirus B19 are the most common causes of myocarditis worldwide.^3^ Coxsackieviruses invade from the enterocytes by binding to the coxsackievirus and adenovirus receptor (CAR), which is located in tight junctional complexes in the intestine.^30 , 31^ This may suggest that pathogens such as coxsackieviruses, which often play a role in the pathogenesis of myocarditis, trigger the development of myocarditis by disrupting the function of tight junctions via CAR receptors located in tight junction complexes, which is regulated via zonulin in the intestinal epithelium and this mechanism may explain the possible increase in intestinal permeability after coxsackievirus infections. Studies have shown that parvovirus B19, one of the most common causes of myocarditis, colonizes intestinal epithelial cells and uses intestinal cells as a reservoir.^32^ Parvovirus B19 has been held responsible for chronic fatigue syndrome by remaining at a reservoir in the intestine,^32^ and it has been emphasized that the increase in intestinal permeability in patients with chronic fatigue syndrome has an important role in the pathogenesis of the disease.^33^ This may shed light on the association of increased intestinal permeability with parvovirus B19 in patients with myocarditis. In addition to classical pathogens such as parvovirus B19 and coxsackievirus, myocarditis cases related to COVID-19 have recently started to be seen frequently with the COVID-19 epidemic.^8 , 9^ Although many pathophysiological mechanisms have been suggested in the development of myocarditis due to COVID-19, the main triggering factor of the disease is not yet known.^8^ Besides affecting many systems, COVID-19 affects the gastrointestinal system frequently.^34^ At the same time, studies have found that intestinal permeability is increased in those who have COVID-19, and high zonulin levels, a biomarker of intestinal permeability, has been found in these patients.^35^ Although the effect of intestinal permeability has not been demonstrated in patients with myocarditis due to COVID-19, the higher zonulin levels observed in myocarditis patients with a recent history of COVID-19, as in our study, may support our idea that COVID-19 triggers the development of myocarditis by increasing intestinal permeability. In addition, although it is seen less frequently than viral agents, the development of myocarditis after gastroenteritis of enteropathogenic bacteria from bacterial causes indicates that impaired intestinal permeability may be associated with the development of myocarditis.^36^

Studies have shown that lipopolysaccharides (endotoxin) increase intestinal permeability.^18^ Presepsin (a soluble cluster of differentiation subtype 14 [CD14]) is a reliable and indirect marker of endotoxemia.^37^ It has been shown to increase in bacterial sepsis and also in viral infections such as COVID-19.^38^ All this shows that presepsin may be an indirect biomarker that can show an increase in intestinal permeability.

In our study, the correlation of high zonulin levels with peak CK-MB and peak troponin-I levels indicates that a greater antigen load triggers greater cardiac destruction as intestinal permeability increases. The fact that zonulin and presepsin levels were high in patients with myocarditis may be epiphenomenal or indicate a complex interaction that plays a role in increased intestinal permeability. The increase in intestinal permeability, confirmed by zonulin levels, may lead to more bacteremia and viremia, which can be explained by an increase in presepsin in correlation with zonulin levels. Furthermore, considering the role of zonulin in the pathogenesis of myocarditis, zonulin may be a potential biomarker for myocarditis, as well as a therapeutic target in severe myocarditis cases, as in other inflammatory diseases.^39^ Enteric viruses utilize gut microbes for replication and transmission,^40^ which may explain why increased intestinal permeability in bacterial gastrointestinal enteritis causes enteric virus transmission and predisposes to myocarditis development. The fact that presepsin and zonulin were found to be high together in myocarditis cases supports this view.

As a result, we hypothesize that increased permeability in the gastrointestinal system may be one of the main triggering mechanisms in the development of myocarditis. Zonulin and presepsin can be used as biomarkers in addition to conventional biomarkers when evaluated by the clinic. This is for the early management of diagnosis and treatment modalities in myocarditis where gold standard diagnostic tools such as EMB and cMRI are unavailable.

If this relationship is clearly demonstrated, early diagnosis and treatment may help prevent chronic complications of myocarditis such as dilated cardiomyopathy. In addition, in cases with similar clinical presentations, the use of these two biomarkers in addition to traditional biomarkers is a cost-effective option in clinical practice by reducing the application of expensive diagnostic methods such as EMB, cMRI and coronary angiography, which are used for the diagnosis and exclusion of acute myocarditis and require special expertise for their application and interpretation of results.

Based on these findings, more extensive studies in this area may shed light on the triggering pathogenesis of myocarditis and assist in the development of new pharmacological models to reduce the likelihood of poor clinical outcomes such as dilated cardiomyopathy.

### Limitations of the study

Our study had some limitations. Firstly, our sample size was relatively small, and it was a cross-sectional study. Secondly, only acute myocarditis patients with high cardiac biomarkers were included in our study. The exclusion of patients with borderline and subclinical myocarditis and patients with chronic myocarditis may affect the results. Patients’ PCR or isolation results of causative viruses could not be included in the study due to technical inadequacy. Still, the combined evaluation of patients’ histories, cMRI results, biological markers such as troponin and CK-MB, and invasive and non-invasive coronary angiographic methods were sufficient to exclude other causes. Finally, endomyocardial biopsy, the gold standard diagnostic tool, was not used in the diagnosis, which may have affected our results.

## Conclusion

Myocarditis may present, similarly to ischemic heart disease, with chest pain, abnormalities in electrocardiograms and elevated cardiac biomarkers. It may be mixed up with these cardiac pathologies that are similar in symptoms and laboratory findings, including cardiac amyloidosis and hypertrophic cardiomyopathy. Therefore, suspected cases of myocarditis remain a challenging diagnosis for clinicians in terms of its presentation, features, and course. In this respect, when gold standard diagnostic tools such as EMB and cMRI are not available, auxiliary biomarkers that can be used in addition to CK-MB and cardiac troponin for rapid diagnosis of myocarditis cases and exclusion of other confounding cardiac causes. In addition, increased respiratory tract and intestinal permeability may be one of the main mechanisms triggering the development of myocarditis. Therefore, zonulin and presepsin may be promising biomarkers for both diagnosis and follow-up treatment in myocarditis patients.
